# Surface Charges at the CaF_2_/Water Interface Allow Very Fast Intermolecular Vibrational‐Energy Transfer

**DOI:** 10.1002/anie.202004686

**Published:** 2020-05-29

**Authors:** Dominika Lesnicki, Zhen Zhang, Mischa Bonn, Marialore Sulpizi, Ellen H. G. Backus

**Affiliations:** ^1^ Institute of Physics, Johannes Gutenberg University Mainz Staudingerweg 7 55099 Mainz Germany; ^2^ Department for Molecular Spectroscopy Max Planck Institute for Polymer Research Ackermannweg 10 55128 Mainz Germany; ^3^ Department of Physical Chemistry University of Vienna Währinger Strasse 42 1090 Vienna Austria

**Keywords:** 2D sum-frequency generation, ab-initio molecular dynamics, energy transfer, solid/liquid interfaces

## Abstract

We investigate the dynamics of water in contact with solid calcium fluoride, where at low pH, localized charges can develop upon fluorite dissolution. We use 2D surface‐specific vibrational spectroscopy to quantify the heterogeneity of the interfacial water (D_2_O) molecules and provide information about the sub‐picosecond vibrational‐energy‐relaxation dynamics at the buried solid/liquid interface. We find that strongly H‐bonded OD groups, with a vibrational frequency below 2500 cm^−1^, display very rapid spectral diffusion and vibrational relaxation; for weakly H‐bonded OD groups, above 2500 cm^−1^, the dynamics slows down substantially. Atomistic simulations based on electronic‐structure theory reveal the molecular origin of energy transport through the local H‐bond network. We conclude that strongly oriented H‐bonded water molecules in the adsorbed layer, whose orientation is pinned by the localized charge defects, can exchange vibrational energy very rapidly due to the strong collective dipole, compensating for a partially missing solvation shell.

## Graphical abstract

**Face the interface**: Using 2D surface‐specific vibrational spectroscopy, the heterogeneity of water molecules at the CaF_2_/water interface is quantified and information about the sub‐picosecond vibrational‐energy‐relaxation dynamics is provided. Strongly oriented H‐bonded water molecules in the adsorbed layer can exchange vibrational energy very rapidly due to a strong collective dipole, compensating for a partially missing solvation shell.

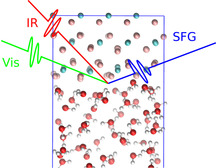

## Introduction

The structural and dynamical properties of water in contact with mineral surfaces are relevant to a wide variety of natural phenomena, for example, the weathering of rocks and corrosions, and to several technological/industrial applications.^1^, ^2^ In particular, the mineral calcium fluorite (CaF_2_)/water interface is central to industrial, environmental, and medical applications, for example, for understanding fluorine dissolution in drinking water. Also, the interfacial properties determine the reactions taking place at the interface.

At the interface, the properties of water are expected to be different from those observed in the bulk, as the H‐bonding network of water is suddenly broken and/or strongly influenced by the solid surface. Indeed, it has been observed that water at the water/air interface can reorient faster than in the bulk^3^ owing to the decreased hydrogen bonding at the interface. The interfacial decrease in H‐bond density has also been invoked to explain the slower energy relaxation at the surface compared to bulk water.^4^ These insights into the dynamics of molecules at the water/air interface were obtained using time‐resolved (tr) and two‐dimensional (2D) sum‐frequency‐generation spectroscopy (SFG).^5^, ^6^, ^7^, ^8^, ^9^ In tr‐ and 2D‐SFG, a significant fraction (≈10 %) of the water molecules will be excited with an intense infrared pulse. The subsequent energy relaxation can be monitored with the SFG‐probe pair, a broadband infrared pulse in resonance with molecular vibrations, and a narrow‐band visible pulse. In these methods, specifically the molecules at the interface are probed, as the SFG selection rule is such that no signal will be obtained from centrosymmetric media like bulk water.^10^ At the interface, the symmetry is broken, resulting in a signal. As such, the (transient) vibrational spectrum of, specifically, the interfacial molecules will be obtained.

For the water/mineral interface, tr‐SFG has also been used to determine relaxation dynamics.^11^, ^12^, ^13^, ^14^, ^15^, ^16^, ^17^ McGuire and Shen^11^ performed the first femtosecond tr‐SFG experiments on the water/silica interface. They found a fast 300 fs time constant for the bleach relaxation. Further experiments by the group of Borguet explored the vibrational dynamics of the silica/water and alumina/water interfaces at different pHs and different ionic strengths. For the silica/water interface, relaxation time scales of H‐bonded water were found to be strongly influenced by the local H‐bond network, by the surface charge, and by the isotopic dilution.^12^, ^13^, ^14^ Recently, it was shown that weakly H‐bonded OH groups of water pointing to SiO_2_ relax on a ps timescale.^17^ For the alumina/water interface, the vibrational‐relaxation dynamics of H‐bonded water were found to be insensitive to surface charge and ionic strength. Moreover, the relaxation dynamics of water at the charged alumina interface is faster than for bulk water.^15^, ^16^ These studies have provided important insights into vibrational lifetimes. While vibrational lifetimes obtained from tr‐SFG can be informative, 2D‐SFG provides, in addition to the vibrational lifetimes, also spectral diffusion dynamics, which can be related to structure, structural dynamics, and interfacial‐energy‐transfer dynamics.^18^

Here we use tr‐ and 2D‐SFG spectroscopy to study the vibrational relaxation and heterogeneity of deuterated water molecules at the CaF_2_/water interface at low pH. We use D_2_O instead of H_2_O, as our experimental setup provides more IR power for both the pump and the probe beam in the O−D stretch region than in the O−H stretch region, and because the dynamics is slower, allowing to follow it with the time resolution of our setup. Moreover, low pH creates, due to fluoride dissolution, local charge defects on the surface, providing a system to study the effect of these localized charges on the water heterogeneity and water dynamics. The experiments are interpreted using non‐equilibrium molecular‐dynamics simulations where the energy relaxation from a vibrationally excited state can be followed and the relaxation time scales can be interpreted in terms of the local environment.^19^ Our approach includes an atomistic description of the interface, where the interatomic potentials are determined by the full electronic structure at the density functional theory (DFT) level. Such models have been able to reproduce the static SFG spectra of the CaF_2_/water interface over a wide pH range.^20^ In the current manuscript, we go beyond the static structure of water at the CaF_2_ interface discussed in our previous work. Here, we use, for the first time, 2D‐SFG at a solid/liquid interface to obtain information about the heterogeneity at the CaF_2_/water interface. Combined with non‐equilibrium MD, we obtain a unique picture about energy dynamics at the CaF_2_/water interface at low pH and the role of localized charge defects.

## Results and Discussion

**Figure 1:**
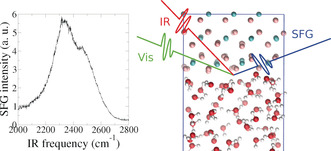
Left: Experimentally recorded static SFG spectrum of D_2_O in contact with CaF_2_ at pD 2. Right: snapshot from the simulation and pictorial view of an SFG experiment.

Before moving to the 2D and time‐resolved spectra, we briefly recall the main features appearing in the static SFG spectra. Figure 1 (left) shows the SFG spectrum of D_2_O at pD=2 in contact with a CaF_2_ prism measured while flowing the aqueous solution. The prism allows experiments in total‐internal‐reflection geometry, enhancing the signal. The response at pD=2 shows an intense broad band between 2200 and 2600 cm^−1^. As detailed in Ref. 20, this band originates from water molecules in the first few Å from the solid surface pointing with their oxygen atoms towards the positively charged CaF_2_ surface.

**Figure 2:**
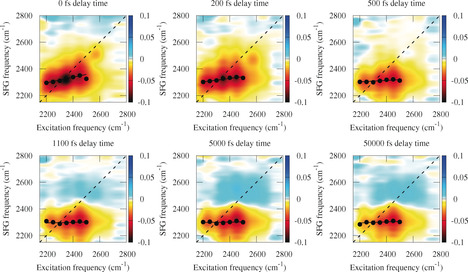
Differential 2D SFG spectrum of D_2_O at pD=2 in contact with CaF_2_ at waiting times of 0, 200, 500, 1100, 5000, and 50 000 fs.

To study the vibrational dynamics and the heterogeneity of water at the fluorite interfaces, we performed 2D‐SFG experiments on this buried solid/liquid interface. Figure 2 shows the collected spectra for D_2_O at pD=2. At early delay times between the IR excitation and the SFG detection, the 2D plot is dominated by a broad negative signal, a bleach, between 2200 and 2500 cm^−1^. Moreover, after 1 ps delay time, a positive signal also appears. The negative–positive feature at long delay times, largely independent of the excitation frequency, reflects the elevated temperature of the system after energy relaxation out of the excited mode. Due to the temperature increase, all hydrogen bonds are slightly weakened, resulting in a blue‐shift of the O−D stretch frequency. This leads to a negative signal at low frequencies and a positive signal at high frequencies. Based on Ref. 21 reporting a temperature increase of 15 K for exciting with 25 μJ IR pulses, we estimate that the temperature increase by up to 5 K.

**Figure 3:**
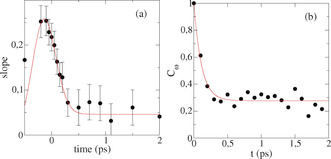
a) Slope of the 2D spectrum of D_2_O at pD=2 in contact with CaF_2_ as a function of delay time between pump and probe (experimental data and fit according to the model reported in the text). b) Frequency–frequency correlation function for the stretching mode as obtained from the simulations (black dots), along with a single‐exponential fit (red line). The resulting spectral diffusion is 100 fs.

At water/air and water/lipid interfaces, the decay of the slope of the 2D‐SFG spectra has been assigned to energy‐transfer processes: the time‐dependent tilt of the bleach signal in a 2D‐ SFG plot in the OH or OD stretch region of pure water, be it H_2_O or D_2_O, provides information about the rate at which vibrational energy is exchanged between the different hydroxyl groups. In case the energy transfer between different water molecules is very slow, one would expect a bleach signal around the diagonal, as, at any given excitation frequency, only that specifically excited sub‐ensemble of OH‐group molecules will respond. On the contrary, in the limit that energy transfer is much faster than the time resolution of the experiments, all water molecules will appear equal, and the response should be rather insensitive to the excitation frequency. As such, a more horizontal bleach is expected. The 2D spectrum for the CaF_2_ interface at early delay times shows a small tilt along the diagonal between 2200 and 2500 cm^−1^, indicating that vibrational‐energy transfer is very rapid. With increasing delay time, the bleach becomes more horizontal due to additional spectral diffusion. The tilt can be quantified by obtaining the slope as graphically depicted in the different panels in Figure 2. The data points are obtained by fitting a Gaussian function through the vertical slices. The black lines are a linear fit through the black points. The time evolution of the slope is reported in Figure 3. From fitting the data with an exponential function convoluted with a Gaussian system‐response function with a full width at half‐maximum (FWHM) of 500 fs (red line in Figure 3), we conclude that the slope decays in less than 100 fs, which is comparable to the decay observed in bulk water,^4^, ^19^ yet substantially faster than what has been observed for the water/air^5^ and water/positively‐charged‐lipid interfaces^22^ with D_2_O as sub‐phase.

The spectral diffusion can also be obtained from the ab‐initio molecular‐dynamics (AIMD) simulations presented in Ref. 20 by calculating the frequency–frequency correlation functions (Figure 3 b). For such a calculation. only the interfacial layer is used with a thickness of 3.5 Å, which is the layer contributing most to the SFG signal according to our previous analysis.^20^ A fast exponential decay on the order of 100 fs is found, in agreement with the experimental results and with the value obtained from the simulations of bulk water in Ref. 19. At first glance, it seems remarkable that interfacial and energy‐transfer dynamics are comparable to that in bulk water, as in the bulk, the density of OH (or, equivalently, OD) groups is much higher. This very fast interfacial energy transfer therefore hints to an enhanced intermolecular coupling at the interface, which could be a result of the surface‐induced alignment of water molecules.

**Figure 4:**
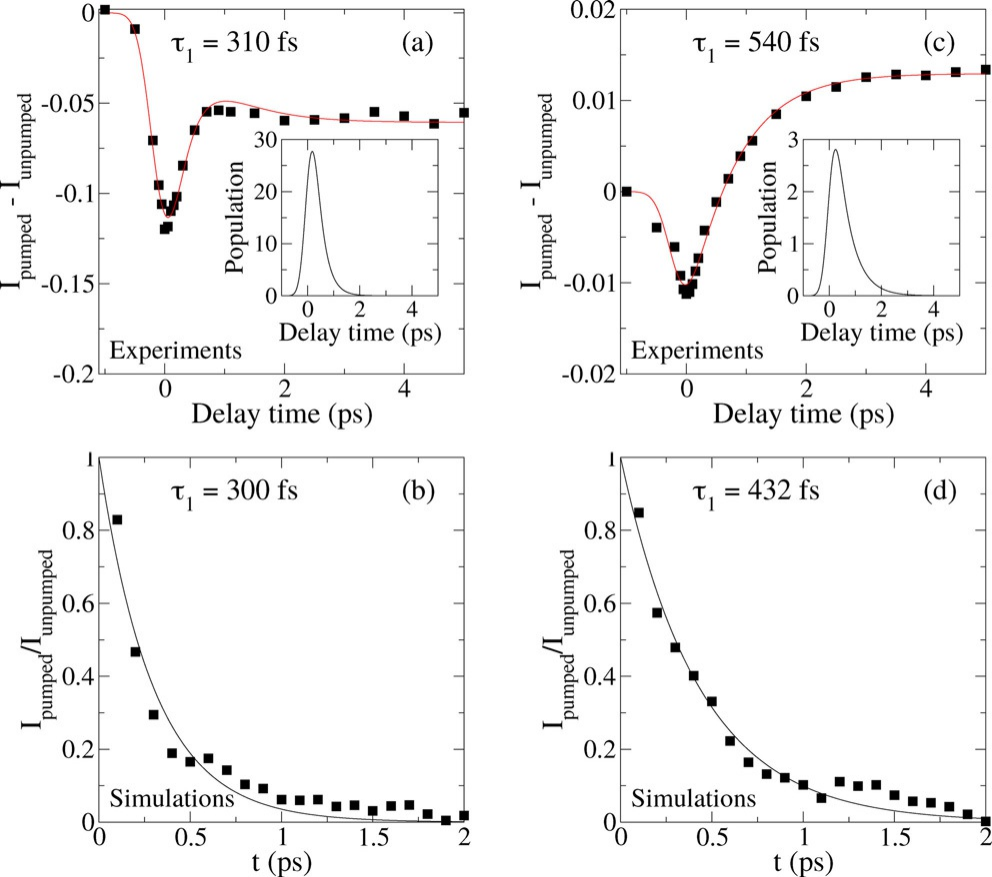
a), c) Difference of SFG‐pumped and unpumped signal for water at pD=2 in contact with CaF_2_ with a pump frequency of a) 2350 and c) 2575 cm^−1^ as a function of time. The black dots are the experimental data obtained by integrating over 60 cm^−1^ around the pump frequency. The red lines are a fit with the four‐level model described in the text. The population of the excited state (×10^−3^) as a function of time is presented in the insets. b), d) Time evolution of the excess energy of the OH stretch obtained from simulations at low pH (square) for the water ensemble with a pump frequency b) below 2515 cm^−1^ and d) above 2515 cm^−1^ normalized by the initial value and its exponential fit (plain).

Besides the time evolution of the slope, the time‐dependent 2D plots also provide information about energy relaxation. To extract this information, we integrated the signals around the diagonal with intervals of roughly 60 cm^−1^. Figure 4 a,c shows examples of these integrated signals around the diagonal for 2350 and 2575 cm^−1^ excitation, respectively, as a function of delay time.

**Figure 5:**
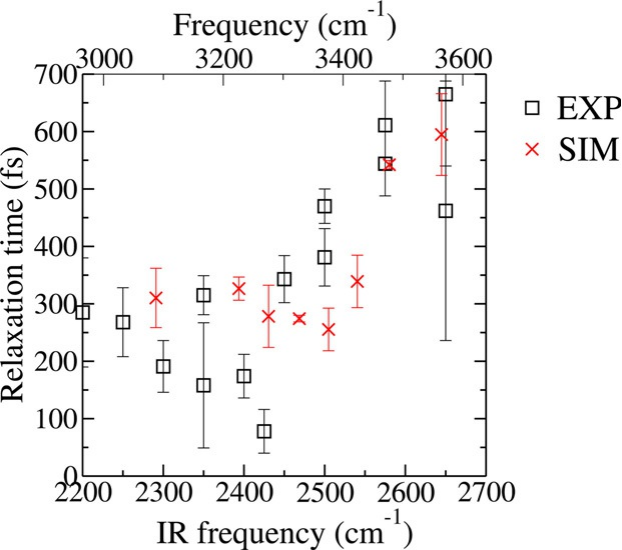
Vibrational‐relaxation time as a function of frequency (black: experimental data; red: simulation).

Both curves show an instantaneous bleach at zero delay time followed by signal relaxation, but not to zero. The negative and positive signals at late times and 2350 as well as 2575 cm^−1^ pumping, respectively, reflect the above‐mentioned weakening of the H‐bond structure due to the elevated temperature of the system after excitation and subsequent relaxation. To obtain timescales for the relaxation, the time traces were modeled with a four‐level model, as is generally used for the relaxation dynamics of water.^22^ Briefly, this model assumes that after excitation from the ground state to the first vibrationally excited state, the system relaxes to an intermediate state often attributed to an overtone of the water‐bending mode from which the system relaxes to the heated ground state. In this relaxation model, two timescales are involved: *τ*_1_ for relaxation to the intermediate level and *τ*_eq_ for relaxation to the heated ground state. The red lines in Figure 4 are fits with this model with a fixed equilibration time of 700 fs, which is taken from bulk water.^23^ The time for relaxation out of the excited state is 310±30 and 540±60 fs for the excitation at 2350 and 2575 cm^−1^, respectively. A detailed frequency dependence of the relaxation time for several points along the diagonal is reported in Figure 5 (black squares).

Similar to the water/air,^4^ water/DPTAP (1,2‐dipalmitoyl trimethylammonium propane),^22^ and silica/water^13^ interfaces, above 2500 cm^−1^, the relaxation time increases with increasing excitation frequency, indicating that the weaker H‐bonded water ensemble is rather heterogeneous. In contrast, below 2500 cm^−1^, this relaxation time is largely frequency‐independent. In combination with the small slope observed in the 2D plot between 2300 and 2500 cm^−1^, this shows that the water molecules below 2500 cm^−1^ form a rather homogeneous ensemble.

For the water/air and water/DPTAP interfaces as well as for bulk water, the frequency dependence of the vibrational lifetime between 2300 and 2550 cm^−1^ (3100 to 3700 cm^−1^ for H_2_O) could be modeled by taking into account a stronger coupling between stretch and bend modes at low frequencies due to a better frequency matching between the bend overtone and the stretch mode.^4^, ^22^ On the contrary, simulations have shown that such heterogeneity in bulk water is related to the local H‐bond structure. A larger number of H‐bonds in the first solvation shell reduces the relaxation time, that is, it speeds up the relaxation.^19^, ^24^, ^25^

The frequency‐independence of the relaxation time for the CaF_2_/water interface at low frequencies hints to a different relaxation mechanism than that previously concluded for bulk water and the water/air as well as water/DPTAP interfaces for which a distinct frequency dependence was observed.^4^, ^22^ Only for the water molecules having a vibrational frequency above 2500 cm^−1^, the mechanism could be similar to water, raising the question of how much bulk water is probed with SFG at high frequencies.

It has recently^26^, ^27^, ^28^, ^29^ been shown that for charged surfaces, the SFG signal consists of a contribution from the layers directly at the interface, the *χ* ^(2)^ signal, and a contribution from the diffuse layer, the so‐called field‐induced *χ* ^(3)^ signal. The ratio between the bulk and surface contributions to the signal depends on the local ion concentration and their ability to screen the surface charge. From salt‐dependent static SFG experiments at the CaF_2_ interface, and taking into account the salt‐induced Debye screening,^26^ we conclude that the bulk fraction at high frequencies is less than 20 %. Therefore, we can conclude that the main contribution to our signal is from the water layers directly at the interface. Please note that this bulk/surface contribution is less of a complication for the phase‐resolved spectra presented in Ref. 20, acquired without a flow cell so that the fluoride dissolution screens the charge at the surface and only surface contributions are measured.

To obtain molecular‐level details of the relaxation mechanism, the experiments were corroborated by simulations of the vibrational relaxation using AIMD simulations according to the method reported in Ref. 19. Starting from the model for the low‐pH interface with 0.64 vacancies nm^−2^ (1 vacancy per surface, that is, 2 per unit cell),^20^ we have selectively excited the water molecules in the first water layer in contact with the surface. Every single excitation is obtained by adding, to a given water molecule, excess kinetic energy in the stretching mode. The excess kinetic energy per molecule is chosen such that the temperature of the overall simulation box is increased by 1.5 K. This is, on the one hand, close to the increment in the temperature of the sample in a typical pump–probe experiment. On the other hand, in this manner, we ensure that the excitation is not strongly perturbing or even disrupting the local H‐bond network.^19^ The excitation spectrum obtained from the simulations is reported in the Supporting Information, Figure S1.

**Figure 6:**
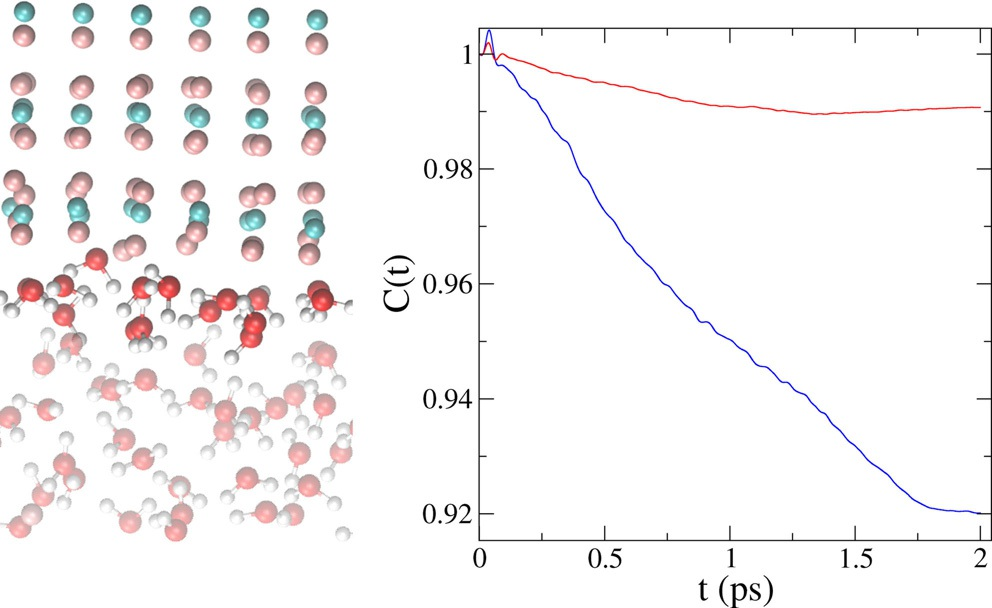
Left: Snapshot of the model used in the simulations. The water molecules in the adsorbed layer and excited in the simulation of the vibrational relaxation are highlighted in full color, while the rest is depicted as transparent. Right: Time evolution of the dipole–dipole correlation function for the adsorbed water in the first layer (red) and for water in the bulk (blue). Data from the bulk are taken from Ref. 19.

Using *NVE* trajectories, we could follow how the excess energy added to the system leaves the original excited water molecule and is redistributed over the system. The relaxation of vibrational energy out of the excited stretching state for excitation frequencies below and above 2515 cm^−1^ are depicted in Figure 4 c,d (for an average excitation frequency of 2400 cm^−1^ and 2590 cm^−1^, respectively). For a more direct comparison with the experimental data, the insets in Figure 4 a,b show the population of the vibrationally excited state obtained by fitting the experimental data with the four‐level model (for an excitation frequency of 2350 cm^−1^ and 2575 cm^−1^, respectively). In Figure 5, the frequency‐dependent relaxation times from the simulations (red crosses), and those from experiments (black squares) are shown together. Like the experiments, the simulations show faster relaxation times at low frequencies and slower relaxation times at higher frequencies. One should be careful when comparing the timescales from simulations and experiments, as the simulations were performed in H_2_O and the experiments in D_2_O. We would expect slightly slower dynamics for D_2_O. To understand the molecular origin of the experimental results, we analyzed the simulations in more detail. To this end, it is interesting to discuss the structure of the adsorbed layer. Such a layer is highlighted in Figure 6 (left), while the rest of the water molecules are rendered as transparent. Different from bulk water, the interfacial layer exhibits a well‐ordered structure dictated by the positive‐charge defects localized in correspondence of the fluoride vacancy. The order extends over 4–5 Å. This was inferred from the calculation from the convergence of the Im(*χ* ^(2)^) spectrum with increasing probing thickness.^20^ We should, however, note here that the high computational cost of electronic‐structure‐based methods imposes severe limitations on the size of the accessible models. In this respect, our model is expected to capture the contribution to the spectra of the Stern layer (possibly the major contribution here), but cannot account for the full diffuse layer, which is expected to extend over a few nanometers thickness. As mentioned above, the experimental data indicate that the contribution of the diffuse layer is small. Close inspection of the interfacial layer reveals that the water molecules directly pinned by the surface defects are frozen. These molecules do not leave their position on the surface during the entire simulation time (an analysis of the root‐mean‐square displacement is reported in the Supporting Information). They only donate two H‐bonds while they do not accept any H‐bonds. Such an order also extends to the other water molecules in the interfacial layer, which, on average, forms only 2.45 H‐bonds (with 62 % donors and 38 % acceptors), which is one H‐bond less compared to bulk water, where molecules form on average 3.48 H‐bonds (with equal acceptor and donor contributions). With respect to bulk water, such molecules have an incomplete first solvation shell. Interestingly though, the interconnecting H‐bond network at the interface is also much more stable, and the intralayer H‐bond dynamics much slower. This can be appreciated by calculating the correlation function between the dipole moment of each water molecule in the layer with the neighboring ones (red curve in Figure 6, right). Such a correlation function is close to one when two molecules maintain their reciprocal orientation as a function of time, namely, when their H‐bond remains intact. On the contrary, the correlation function decays to zero if the reciprocal orientation is lost or, in other words, if the H‐bond is broken. For the water in the first adsorbed layer on the surface, the dipole–dipole correlation function (red curve in Figure 6, right) decays very slowly when compared to the same dipole–dipole correlation function calculated for bulk water (blue curve). The simulations reveal a quite interesting scenario, where the interfacial water molecules present a strongly ordered and asymmetric H‐bond network. Relative to bulk water, the interfacial water molecules are missing almost half of their solvation shell. However, the increased order, produced by the localized positive charges on the surface, apparently compensates for the missing H‐bonds, enabling fast vibrational‐energy transfer despite the lower effective water density. This finding is in line with the case of ice I_h_, in which strong ordered bonds can lead to relaxation‐time constants as short as 80 fs,^30^ which could be a result, for example, of the higher stretching‐mode delocalization.

## Conclusion

2D and time‐resolved SFG spectra of the buried CaF_2_/water interface showed that water molecules with a relatively strong intermolecular H‐bond form a rather homogeneous ensemble with very fast relaxation. Weaker H‐bonded water molecules, vibrating at higher frequencies, have a slower vibrational relaxation. We have demonstrated that extremely fast energy transfer can be achieved with only half a solvation shell when the water molecules are strongly oriented by the presence of localized charge defects and, therefore, form very strong and stable hydrogen bonds with their decreased solvation shell. For ordered OH groups and strong H‐bonds, the energy transfer within half a shell may be as efficient as through a full solvation shell in bulk water. This, once more, points to the need for treating water interactions at interfaces with special care, where in response to the specific surface properties, water can show a very peculiar behavior with strong implications for the local reactivity.

## Conflict of interest

The authors declare no conflict of interest.

## Supporting information

As a service to our authors and readers, this journal provides supporting information supplied by the authors. Such materials are peer reviewed and may be re‐organized for online delivery, but are not copy‐edited or typeset. Technical support issues arising from supporting information (other than missing files) should be addressed to the authors.

Supplementary
