# Supplementary material for: Surface Charges at the CaF2/Water Interface Allow Very Fast Intermolecular Vibrational‐Energy Transfer
Source: Angew Chem Int Ed Engl. 2020 May 29;59(31):13116–21. doi: 10.1002/anie.202004686 (PMC7496624; doi:10.1002/anie.202004686)
Supplement: Supplementary file 1 — Supplementary [file ANIE-59-13116-s001.pdf]

Angewandte Chemie
Eine Zeitschrift der Gesellschaft Deutscher Chemiker

Supporting Information

Surface Charges at the CaF_2/Water Interface Allow Very Fast Intermolecular Vibrational-Energy Transfer

Dominika Lesnicki[+], Zhen Zhang[+], Mischa Bonn, Marialore Sulpizi, and Ellen H. G. Backus**

anie_202004686_sm_miscellaneous_information.pdf

1 Time-Resolved 2D-SFG

The SFG setup was based on a Ti:sapphire femtosecond amplifier (Spitfire Ace, Spectra-Physics) pumped by two Nd:YLF (neodymium-doped yttrium lithium fluoride) lasers (Empower, Spectra-Physics) and seeded by the output of an oscillator (Mai Tai, Spectra-Physics). The resulting laser pulses have an energy of 10mJ centred around 800 nm and a 40 fs duration at a repetition rate of 1 kHz. 3mJ and 1mJ of the laser output were used to pump two commercial optical parametric amplifiers (TOPAS-C, Light Conversion). The signal and idler output of the TOPAS pumped with 3mJ were difference frequency mixed in a silver gallium disulfide (AgGaS2) crystal, resulting in at the sample position 6.5mJ IR pulses centered at 2500 cm^{-1} with a full width at half maximum (FWHM) of $\sim$450 cm^{-1}. The narrow band visible upconversion pulses (10 μJ; FWHM, $\sim$15 cm^{-1}) were obtained by passing another 2mJ of the laser output through a Fabry-Perot etalon (SLS Optics Ltd.). The IR and visible probe beams were spatially and temporally overlapped on the sample with incident angles of 40° and 70°, respectively, with respect to the surface normal. The

reflected SFG signal is detected with a spectrograph (Acton, SpectraPro-300i) and EMCCD camera (Newton, Andor).

In the SFG-2D-IR experiment, an additional high intensity and narrow-band pump pulse is used to excite ground state molecules to the vibrational excited state. This pulse is generated by parametric amplifying the doubled idler centered around 1000 nm with 2mJ of the 800 nm laser output in a $LiNbO_3$ crystal. The doubled idler is obtained by passing the output of the TOPAS pumped with 1mJ through a BBO crystal. The resulting pump pulses were tuned between 2200 and 2800 cm^{-1} and have an energy at the sample between 1 and 8 μJ with an incident angle of 56 ^{o}. The bandwidth is typically around 100 cm^{-1}.

The IR pump, IR probe, and VIS pulses propagated in one vertical plane. The polarization of the probe SFG/VIS/IR beams were controlled using λ /2 plates and polarizers and were S/S/P in all experiment. The polarization of the pump pulses was P. The spatial overlapped of all three incoming beams was optimized by the third-order cross correlation generated from a gold coated prism and from the CaF_2/D_2O interface as well. Time zero was also defined by the cross correlation. The fit with the model explained in the text allowed a small shift in the zero time.

2D-SFG spectra are obtained by subtracted pumped and unpumped spectra at several pump frequencies. The pump and unpumped spectra are measured semi-simultaneously with the help of a mechanical chopper synchronized with a vibrating galvano-mirror working at 500 Hz. In this way, a signal with excitation pulse on (pumped) and without excitation (unpumped) pulse are separated on the CCD camera.

The setup was flushed with N_2 gas to avoid absorption of the IR light by CO_2 in the air.

2 Sample cell

The experimental sample flow cell consists of a CaF_2 prism (equilateral, 30 mm, Korth Kristalle GmbH) pressed against a teflon cell sealed with a viton O-ring. To avoid local heating the liquid is flown by a peristaltic pump underneath the prism. The circuit contains a teflon reservoir and Tygon chemical tubing. The teflon parts were cleaned with piranha solution (3:1 volume ratio of sulfuric acid and 30% hydrogen peroxide solution). The CaF_2 prism was periodically cleaned by quickly dipping the prism in a solution of concentrated sulfuric acid containing NoChromix, followed by copious rinsing in water. After prolonged exposure to the aqueous phase, especially under extreme pH conditions, the prism was polished using 1 μm and 1/4 μm diamond polishing compounds and Microcloth polishing cloths (Buehler). Polishing was always followed by H_2SO_4/NoChromix cleaning and copious water rinses to remove any surface active contaminants and bake it in the oven at 500°C for 5 hours. To check the cleanliness of the prism the C-H SFG spectra has been checked; no contamination has been observed.

3 Chemicals

The pD=2.4 solution was prepared by dissolving DCl into D_2O. Both D_2O (Cambridge Isotope Laboratories. Inc., 99.96 atom %D) and the salt DCl (Sigma-Aldrich, purity >99.0%) were used without further purification.

4 Data analysis

2D-SFG spectra at different delay times are constructed from the pump-probe experiments measured as function of time delay between pump and probe pulses by merging difference spectra measured at different pump frequencies together. The slope in the 2D plots is obtained by fitting a Gaussian lineshape through the vertical slices. The dots in the 2D plots mark the central frequency of the Gaussian. Lifetimes are obtained by integrating selected parts of the 2D spectrum and subsequent fitting of a four level model as explained in the main text.

5 Simulation setup

We used the same simulation set up used in previous work[1], where the low pH model is the one with a concentration of fluorite vacancies on the surface of 0.64 vacancies.nm^{-2}. The interface between CaF_2 (111) and water is composed of 88 water molecules and 60 formula units of CaF_2 contained in a 11.59Å x 13.38Å x 34.0Å cell periodically repeated in the (x, y, z) directions. The thickness of water slabs is around 20Å along the z-axis, which is reasonable compromise between the need to achieve bulk-like properties far from the surface and the computational cost. All the simulations have been carried out with the package CP2K/Quickstep[2], consisting in Born-Oppenheimer MD (BOMD) BLYP[3, 4] electronic representation including Grimme (D3) correction for dispersion[5], GTH pseudopotentials[6, 7], a combined Plane-Wave (280 Ry density cutoff) and TZV2P basis sets. For the analysis of the vibrational energy relaxation we used the same approach presented in our previous work [8]. As a first we ran a long AIMD simulation in the NVT ensemble with the Nose-Hoover thermostat where a timestep of 0.5 fs for integrating equation of motion was used. From this trajectory 82 snapshots were extracted every 5 ps in order to ensure that velocities are decorrelated. These snapshots were used as the initial coordinates and velocities for the nonequilibrium AIMD runs. The average temperature in the NVT AIMD simulation was 330 K in order to reproduce the water liquid structure at 1 g/cm^3 [9]. We then ran 2 ps AIMD simulations with a timestep of 0.1 fs for the excited and not excited case in the NVE ensemble. Excitations were performed for the water molecules located within 3Å away from the surfaces (composed of fluorine), leading to a total of 552 excitations. We excite each single water molecule such that the temperature of the simulation box is increased by 1.5 K. This is close to the increment in the temperature of the

sample which is measured in the experiments and also ensures that the excitation is not strongly perturbing, or even disrupting, the local hydrogen bond network. Fig 1 shows the frequency distribution obtained from the simulations. The extra kinetic energy, added to a given molecule, drives, instantaneously, the system out of the equilibrium and the subsequent relaxation process can be followed within NVE trajectories using descriptors, which we introduced in Ref.[8] and which permit to follow the excess energy redistribution among the given modes. The time evolution of the excess vibrational energy can be obtained as difference between the power spectrum calculated in the vibrationally excited (or non equilibrium) state and that calculated in the ground (or equilibrium) state, according to:

$$I(t) = \int_0^\infty P^{ex}(\omega, t)\ d\omega - \int_0^\infty P^{gs}(\omega, t)\ d\omega. \tag{1}$$

where

$$P(\omega, t) = \int_t^{t+\Delta t} \left\langle \sum_{ij} \mathbf{v}_i(t)\mathbf{v}_j(t+\tau) \right\rangle e^{-i\omega\tau}\ d\tau. \tag{2}$$

is the Fourier transform of the velocity-velocity autocorrelation function calculated at a given time t and $\mathbf{v}_i(t)$ is the velocity of atom i at time t and Δt the time window over which the correlation function is computed (see Supporting Information for [illegible]

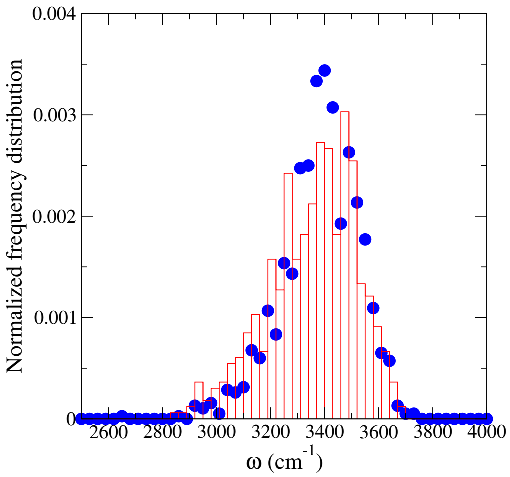

Figure 1: Normalized distribution of the stretch frequencies of the water located in the first layer (red) and in the bulk (blue) [8] at t=0.1 ps.

6 Spectral diffusion from equilibrium and non-equilibrium simulations

The spectral diffusion can be directly obtained in the simulations from the time decay of the frequency-frequency correlation function (FFCF) defined by

$$C_\omega(t) = \langle \delta\omega(t)\delta\omega(0)\rangle \,/\, \langle \delta\omega(0)^2 \rangle \qquad (3)$$

where $\delta\omega(t) = \omega_{max}(t) - \bar{\omega}_{max}(t)$ is the fluctuation from the average frequency at time t. $C_\omega(t)$ can be calculated both in equilibrium, as well as in non-equilibrium simulations, namely following the vibrational excitation. The results of the frequency-frequency correlation are shown in Fig. 2 for both the equilibrium case (black dots) and the non equilibrium case (red dots). For both cases a fast exponential decay of 140 fs and 100 fs, respectively, are found in agreement with the experiments and the value reported for bulk water[10, 8]. For such calculations only the water molecules in the interfacial layer with a thickness of 3.5Å are included, which are those contributing most to the SFG signal, according to our previous analysis[1].

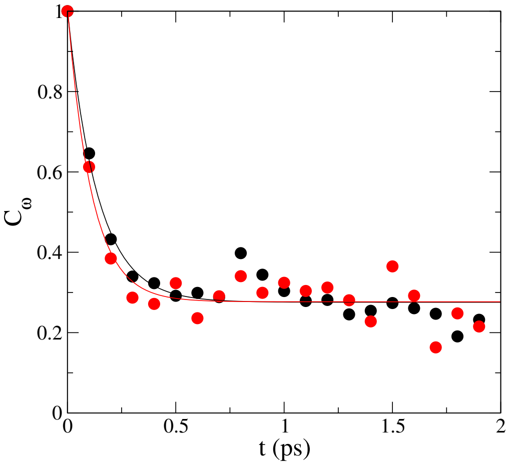

Figure 2: Frequency-frequency correlation function as function of time for both the equilibrium case (black dots) and the non equilibrium case (red dots). A single exponential fit provides a lifetime of 140 fs and 100 fs for the equilibrium (black curve) and non equilibrium (red curve) cases, respectively.

7 Mean square displacements

To quantify the dynamical properties of the water molecules in different environments, interfacial water and bulk, we have computed the mean square

displacement (MSD). The MSD is defined from the trajectories $\vec{r}_i(t)$ of the diffusing particles, labelled with the index i, in terms of the distance from their initial position $\vec{r}_i(0)$:

$$\left\langle \Delta\vec{r}(t)^2 \right\rangle = \frac{1}{N_w} \left\langle \sum_{i=1}^{N_w} |\vec{r}_i(t) - \vec{r}_i(0)|^2 \right\rangle \tag{4}$$

where N_w is the number of water molecules considered. For the calculation of the MSD, we have used a sliding window of 10 ps over 50 ps trajectories of water at CaF_2 interface [1] and bulk water [8]. For the former trajectory, we have selected water molecules up to 3.5Å from the interface. The diffusion coefficient, D, was then computed using the Einstein relation:

$$D = \frac{1}{6} \lim_{t\to+\infty} \frac{d}{dt} \left\langle \Delta\vec{r}(t)^2 \right\rangle \tag{5}$$

The water molecules in the interfacial layer diffuse slower, D = 2.197 10^{-10},

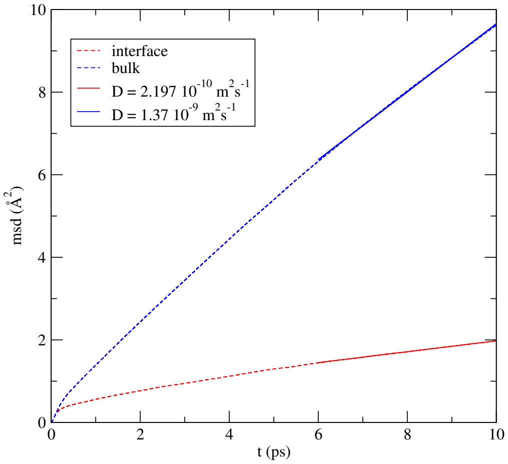

Figure 3: Mean square displacement of the water molecules in the first layer (red) and for water in the bulk (blue). The plain lines display the linear fit used to compute the diffusion coefficient.

than the water molecules in bulk water, D = 1.37 10^{-9} (see Fig. 3).

8 Population

The insets in figure 4 of the main manuscript are shown in Fig. 4.

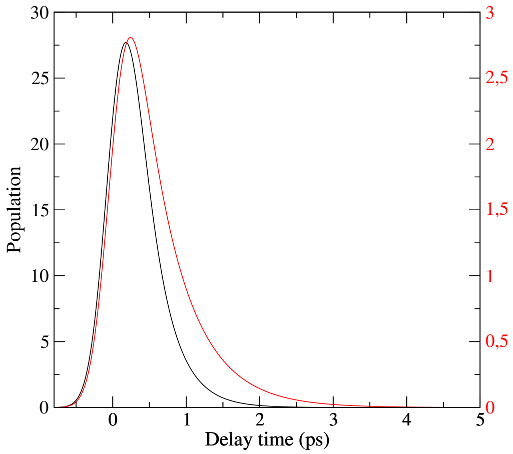

Figure 4: Polulation of the excited state ($\times 10^{-3}$) as function of time for a pump frequency of 2350 cm^{-1} (black) and 2575 cm^{-1} (red).

References

[1] R. Khatib, E. H. G. Backus, M. Bonn, M.-J. Perez-Haro, M.-P. Gaigeot, M. Sulpizi, *Scientific Reports* **2016**, *6*, 24287.

[2] J. VandeVondele, M. Krack, F. Mohamed, M. Parrinello, T. Chassaing, J. Hutter, *Computer Physics Communications* **2005**, *167*, 103 – 128.

[3] A. D. Becke, *Phys. Rev. A* **1988**, *38*, 3098–3100.

[4] C. Lee, W. Yang, R. G. Parr, *Phys. Rev. B* **1988**, *37*, 785–789.

[5] J. VandeVondele, M. Krack, F. Mohamed, M. Parrinello, T. Chassaing, J. Hutter, *Computer Physics Communications* **2005**, *167*, 103 – 128.

[6] S. Goedecker, M. Teter, J. Hutter, *Phys. Rev. B* **1996**, *54*, 1703–1710.

[7] C. Hartwigsen, S. Goedecker, J. Hutter, *Phys. Rev. B* **1998**, *58*, 3641–3662.

[8] D. Lesnicki, M. Sulpizi, *The Journal of Physical Chemistry B* **2018**, *122*, 6604–6609.

[9] J. Schmidt, J. VandeVondele, I.-F. W. Kuo, D. Sebastiani, J. I. Siepmann, J. Hutter, C. J. Mundy, *The Journal of Physical Chemistry B* **2009**, *113*, 11959–11964.

[10] S. T. van der Post, C.-S. Hsieh, M. Okuno, Y. Nagata, H. J. Bakker, M. Bonn, J. Hunger, *Nature Communications* **2015**, *6*, 8384.
